# Integration of Non-Invasive Micro-Test Technology and ^15^N Tracing Reveals the Impact of Nitrogen Forms at Different Concentrations on Respiratory and Primary Metabolism in *Glycyrrhiza uralensis*

**DOI:** 10.3390/ijms27010317

**Published:** 2025-12-27

**Authors:** Ying Chen, Yisu Cao, Yuan Jiang, Yanjun Wang, Zhengru Zhang, Yuanfan Zhang, Zhirong Sun

**Affiliations:** 1 School of Chinese Materia Medica, Beijing University of Chinese Medicine, Beijing 102488, China; chenying578119815@163.com (Y.C.); caoyisu0709@163.com (Y.C.); 2022094131@bucm.ac.cn (Y.J.); 20220935153@bucm.ac.cn (Y.W.); 20210935142@bucm.ac.cn (Z.Z.);; 2 Faculty of Pharmacy, Fujian University of Traditional Chinese Medicine, Fuzhou 350122, China

**Keywords:** *Glycyrrhiza uralensis*, nitrogen forms, respiration, NMT, nitrogen metabolism, primary metabolism

## Abstract

*Glycyrrhiza uralensis* is a highly valued medicinal species worldwide. However, a paradox arises in its cultivation in that high nitrogen fertilization boosts yield at the expense of root quality, a problem linked to nitrogen’s regulation of tricarboxylic acid (TCA) cycle-driven respiration. It remains unclear how different nitrogen forms coordinate respiratory and primary metabolism. We examined the regulatory mechanisms of nitrate (NO_3_^−^) versus ammonium (NH_4_^+^) on these processes in cultivated *G. uralensis* by supplying seedlings with varying concentrations of K^15^NO_3_ or (^15^NH_4_)_2_SO_4_ in a modified Hoagland solution (HNS). Using non-invasive micro-test technology (NMT) and ^15^N tracing, we found that *G. uralensis* employs distinct nitrogen acquisition strategies: sustaining uptake at optimal NH_4_^+^ and low-to-moderate NO_3_^−^, while declining uptake under high NO_3_^−^. These strategies drove form-specific differences in the activity of key nitrogen assimilation enzymes, nitrate reductase and nitrite reductase (NR/NiR), as well as glutamine synthetase and glutamate synthase (GS/GOGAT), and subsequent glutamate and glutamine accumulation. Ammonium nutrition enhanced primary ammonia assimilation and gamma-aminobutyric acid (GABA) metabolism, leading to greater glutamate and endogenous GABA levels. In contrast, nitrate nutrition preferentially stimulated the TCA cycle, resulting in higher accumulation of *α*-ketoglutarate (KGA) and succinate. The concomitant increase in GABA catabolism supported this nitrogen-responsive respiratory metabolism, acting as a compensatory mechanism to maintain KGA homeostasis. Our findings inform nitrogen form strategies for *G. uralensis* cultivation.

## 1. Introduction

*Glycyrrhiza uralensis Fisch.*, commonly referred to as Chinese licorice, is a leguminous plant and ranks among the most economically significant medicinal plants worldwide [1]. Its roots and rhizomes are rich in bioactive compounds like glycyrrhizin, which exhibits notable anti-inflammatory, antiviral, and gastroprotective activities, underpinning their extensive use in medicine, as a natural sweetener, and in the food industry [2,3]. The increasing market demand for *G. uralensis*, alongside the limited availability of wild resources, has driven the necessity for its cultivation [4]. *G. uralensis*, indigenous to the nitrogen-deficient soils of semi-arid Asia, naturally thrives in environments that stimulate the accumulation of its valuable secondary metabolites [5]. However, conventional cultivation often employs high nitrogen fertilization to boost yield, which contrasts with its ecological adaptation, thereby compromising the quality of the final product [6], posing a major challenge to the sustainable development of its industry. Therefore, a comprehensive understanding of nitrogen utilization in *G. uralensis* under varying nitrogen supplies is critical for developing effective nitrogen management strategies to enhance the medicinal quality of its cultivated varieties.

Nitrogen is an essential macronutrient and a fundamental component of amino acids, proteins, and other vital biomolecules, as well as a key environmental factor that orchestrates plant growth and physiological metabolic processes [7]. Plants primarily absorb nitrogen from the soil in the inorganic forms of ammonium (NH_4_^+^) and nitrate (NO_3_^−^), which are channeled into a critical metabolic pathway encompassing their uptake, transport, and assimilation into amino acids [8]. Root cells absorb these ions through specific transporter families (ammonium transporters *AMTs* and nitrate transporters *NRTs*), a process that generates ion fluxes across the root surface and is regulated at the transcriptional level by nitrogen availability [9]. Deciphering these processes requires techniques that capture both the immediate kinetics and the integrated fate of nitrogen.

Non-invasive Micro-test Technology (NMT) permits the direct, real-time, and in situ measurement of dynamic ion fluxes in living plant tissues, providing unique insights into the electrophysiological dynamics of nitrogen acquisition at the root surface [10,11]. Concurrently, ^15^N isotopic tracing serves as an effective method for precisely tracking the uptake, translocation, and allocation of nitrogen within plants, capitalizing on natural isotopic discrimination [12]. These methodologies have been successfully applied in both agricultural and forestry research to study nitrogen utilization efficiency and allocation patterns [8,13,14]. Following uptake, NO_3_^−^ is transported and reduced to NH_4_^+^ through the sequential catalysis of nitrate reductase (NR) and nitrite reductase (NiR). The resulting NH_4_^+^ is subsequently assimilated into amino acids primarily via the glutamine synthetase/glutamate synthase (GS/GOGAT) cycle, with possible contribution from the glutamate dehydrogenase (GDH) pathway [15]. Moreover, nitrogen metabolism provides not only the nitrogen-containing compounds essential for life but also supplies precursors for a wide range of intermediate metabolites and secondary products. Among these are plant flavonoids and phenolic compounds, which originate from phenylalanine, an amino acid whose biosynthesis is intimately connected to central nitrogen assimilatory pathways [16,17]. Our prior research has demonstrated that low-level NH_4_^+^ and NO_3_^−^ treatment positively regulated secondary metabolism, especially the biosynthesis of flavonoids in *G. uralensis* [5]. Yet, a comprehensive insight into the initial steps of NH_4_^+^ and NO_3_^−^ uptake and assimilation in response to different nitrogen forms in *G. uralensis* remains elusive.

Critically, nitrogen assimilation is energetically expensive and intrinsically linked to carbon metabolism [18]. Glutamate serves as a critical entry point for inorganic nitrogen into protein metabolism and represents a key node linking carbon and nitrogen metabolic pathways. It can be decarboxylated by glutamate decarboxylase (GAD) into gamma-aminobutyric acid (GABA), which interfaces directly with the tricarboxylic acid (TCA) cycle via the GABA shunt. In this pathway, GABA transaminase (GABA-T) and succinic semialdehyde dehydrogenase (SSADH) sequentially convert GABA into succinate, thereby directly feeding this TCA cycle intermediate and generating nicotinamide adenine dinucleotide phosphate (NADPH) to support respiratory metabolism. In plants, numerous studies have revealed that GABA is involved in multiple vital biological processes, including plant growth and development, carbon/nitrogen homeostasis, and stress responses, and is closely associated with respiratory metabolism [19,20]. Preliminary evidence suggests a strong relationship between respiratory metabolism and the accumulation of bioactive components in *G. uralensis* under different nitrogen forms [5]. However, an integrated view of how NH_4_^+^ and NO_3_^−^ influence the continuum from root nitrogen uptake and assimilation to its channeling into respiratory pathways has not been fully elucidated.

To decipher the interplay between nitrogen, respiratory, and primary metabolism in *G. uralensis* under different nitrogen forms, we undertook an integrated approach focusing on seedlings supplied with low, medium, and high levels of NH_4_^+^ or NO_3_^−^. Our strategy combined ^15^N isotopic labeling for tracking nitrogen fate with NMT to resolve real-time NH_4_^+^/NO_3_^−^ dynamics at the cellular level. Concurrently, we measured growth parameters, total carbon/nitrogen content, and soluble proteins to assess C-N biomass allocation. To directly link nitrogen assimilation with respiratory metabolism, we analysed respiratory rates, key TCA cycle intermediates, and the activity of key nitrogen-assimilating enzymes. By synthesising these findings, this study elucidates the mechanistic basis of nitrogen form effects on respiratory and primary metabolism in *G. uralensis*, offering new insights for informing fertilization practices in *G. uralensis* cultivation.

## 2. Results

### 2.1. Growth Characteristics of G. uralensis Under Different Nitrogen Sources

**Figure 1:**
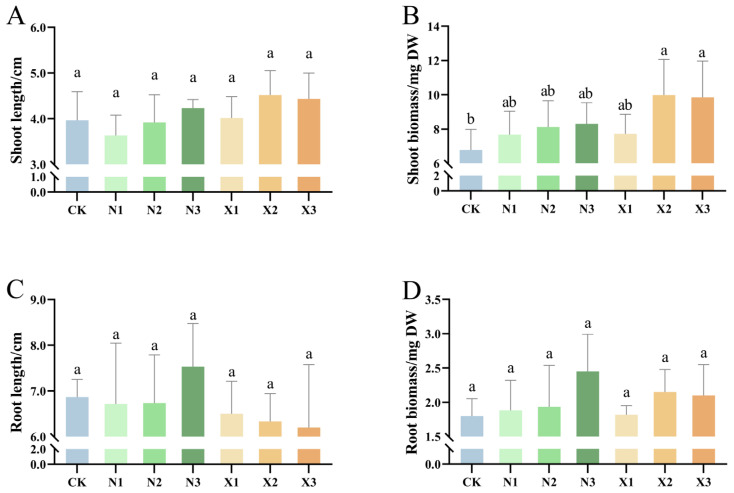
Growth characteristics of *G. uralensis* in response to different nitrogen sources: modified Hoagland solution (Control, CK); 0.25, 0.5, 1.25 mmol/L (^15^NH_4_)_2_SO_4_ (treatments N1, N2, N3); 0.5, 1.0, 2.5 mmol/L K^15^NO_3_ (treatments X1, X2, X3), including shoot length (**A**), shoot biomass (**B**), root length (**C**), and root biomass (**D**). Different letters in the same column indicate a significant difference (*p* < 0.05). Dry Weight: DW.

The most pronounced effect of nitrogen form was on shoot dry weight, which was higher in nitrate-treated plants than in ammonium-treated plants (Figure 1B). Specifically, the X2 treatment produced the greatest shoot biomass, with a significant 47.17% increase over the control. However, shoot length, root length, and root dry weight of *G. uralensis* showed no significant alterations across all nitrogen treatments relative to the control (Figure 1 and Figure S1).

### 2.2. Changes in Root Respiration

**Figure 2:**
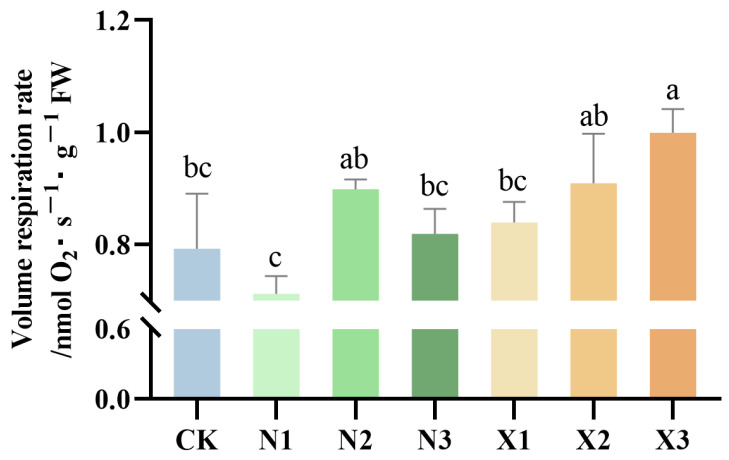
Changes in the root respiration of the *G. uralensis* with different nitrogen forms supplied: modified Hoagland solution (control, CK); 0.25, 0.5, 1.25 mmol/L (^15^NH_4_)_2_SO_4_ (treatments N1, N2, N3); 0.5, 1.0, 2.5 mmol/L K^15^NO_3_ (treatments X1, X2, X3). Different letters indicate a significant difference (*p* < 0.05).

The root volume respiration rate responded differently to the two nitrogen forms. Under ammonium treatments, the root volume respiration rate varied relative to the control, being lower in N1 but higher in N2 (Figure 2). In contrast, a dose-dependent increase in root volume respiration rate was observed in response to elevating nitrate concentrations. The highest rate was observed under the X3 treatment, which showed a significant 26.03% increase compared to the control.

### 2.3. Net Fluxes of NH_4_^+^ and NO_3_^−^

**Figure 3:**
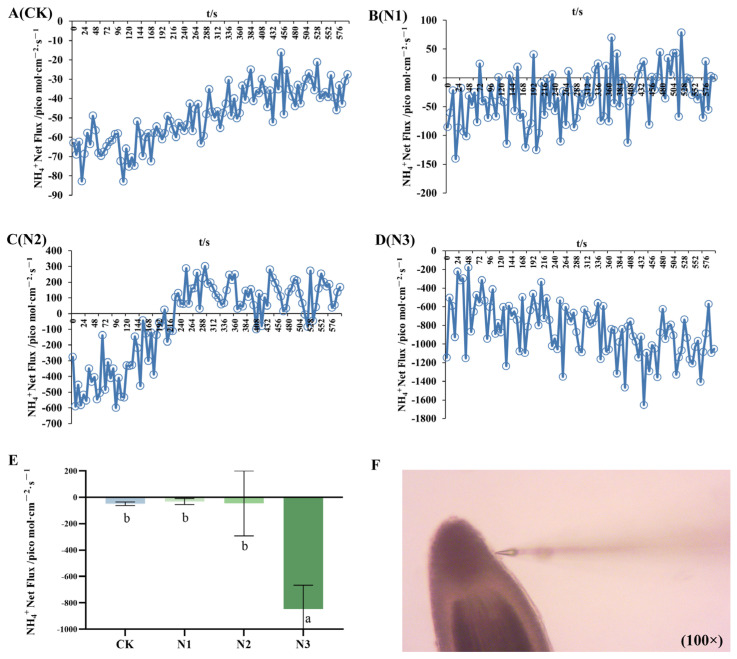
NH_4_^+^ dynamic net flux rates in the root of the *G. uralensis* under control and ammonium treatments: control (CK, (**A**)) in modified Hoagland nutrient solution; ammonium treatments: 0.25, 0.5, and 1.25 mmol/L (^15^NH_4_)_2_SO_4_ (labeled N1, N2, and N3, panels (**B**), (**C**), and (**D**), respectively). NH_4_^+^ net flux rates (**E**). Morphology and root tip site NH_4_^+^ net flux rates were monitored using a non-invasive micro-test (NMT): (**F**). Positive values indicate ion efflux; negative values indicate ion influx. Bars labelled with different letters indicate a significant difference between the treatments. (*p* < 0.05).

The application of Non-invasive Micro-test Technology (NMT) enabled the determination of dynamic ion fluxes in *G. uralensis* roots in response to different nitrogen forms, providing direct insight into the absorption dynamics. CK exhibited continuous NH_4_^+^ influx, with the fluctuation in uptake rate gradually diminishing over time. N1 showed slight NH_4_^+^ efflux, while the N2 treatment resulted in dynamic alternation between NH_4_^+^ influx and efflux. In contrast, the N3 treatment induced sustained NH_4_^+^ influx, with uptake rates ranging from 177.584 to 1653.749 pmol·cm^−2^·s^−1^ (Figure 3). Overall, *G. uralensis* roots demonstrated net NH_4_^+^ absorption, and the rate of uptake increased with higher ammonium nitrogen concentration. With NH_4_^+^ as the sole N source, the *G. uralensis* seedlings cultured in different concentrations of ammonium nitrogen displayed a general NH_4_^+^ influx, with no significant difference in the NH_4_^+^ influx rates between N1 and N2. However, with high N supply, the net NH_4_^+^ influx rate was 17.32-fold compared to the control (Figure 3E).

**Figure 4:**
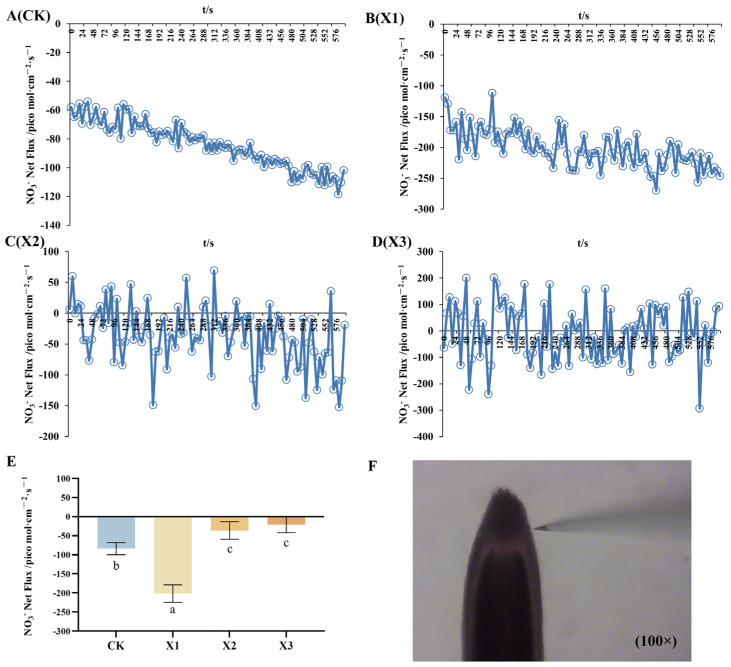
NO_3_^−^ dynamic net flux rates in the root of the *G. uralensis* under control and nitrate treatments: control (CK, (**A**)) in modified Hoagland nutrient solution; nitrate treatments: 0.5, 1.0, and 2.5 mmol/L K^15^NO_3_ (labeled X1, X2, and X3, panels (**B**), (**C**), and (**D**), respectively). NO_3_^−^ net flux rates (**E**). Morphology and root tip site NO_3_^−^ net flux rates were monitored using a non-invasive micro-test (NMT): (**F**). Positive values indicate ion efflux; negative values indicate ion influx. Bars labelled with different letters indicate a significant difference between the treatments. (*p* < 0.05).

Roots in the control group exhibited continuous dynamic uptake of NO_3_^−^ ions. Under the X1 treatment, NO_3_^−^ flux rates varied between 111.601 and 256.510 pmol·cm^−2^·s^−1^. In the X2 group, the NO_3_^−^ flux transitioned from fluctuating efflux to fluctuating absorption over time, while the X3 treatment showed alternating phases of NO_3_^−^ influx and efflux, peaking at 238.856 and 200.124 pmol·cm^−2^·s^−1^, respectively. These results indicated that as the concentration of nitrate nitrogen increases, NO_3_^−^ ion dynamics in *G. uralensis* roots shift gradually from net absorption toward efflux (Figure 4). Consistent with this trend, under sole nitrate nitrogen supply, a pronounced NO_3_^−^ influx was observed in *G. uralensis* seedlings (Figure 4E). Notably, the net NO_3_^−^ influx rate under low NO_3_^−^ supply (X1) was 2.40 times higher than that of the control. However, the net NO_3_^−^ influx rate showed no significant difference between the moderate and low nitrate treatment groups, both of which were lower than the control group, while the high nitrate treatment group exhibited the lowest rate.

### 2.4. Contents of Total N, Total C, and δ^15^N

**Figure 5:**
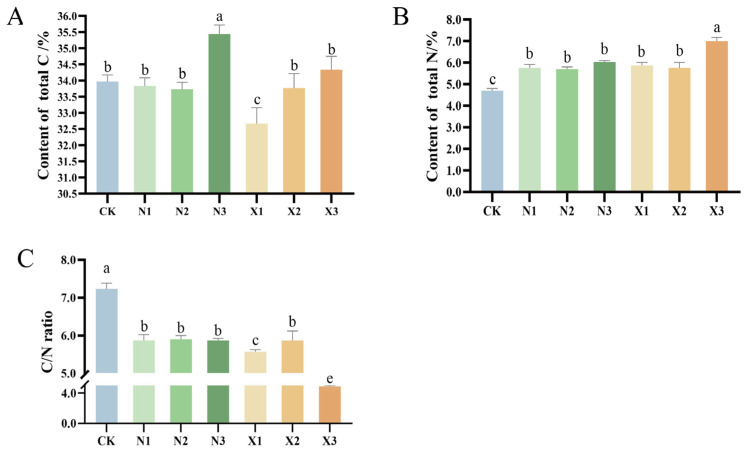
Content of total C (**A**), total N (**B**) and C/N ratio (**C**) in the root of the *G. uralensis* with different nitrogen forms supply: modified Hoagland solution (control, CK); 0.25, 0.5, 1.25 mmol/L (^15^NH_4_)_2_SO_4_ (treatments N1, N2, N3); 0.5, 1.0, 2.5 mmol/L K^15^NO_3_ (treatments X1, X2, X3). Different letters above bars denote significant differences among treatments (*p* < 0.05).

High ammonium nitrogen supply increased the total C concentration in the roots. The total carbon content of N3 was significantly higher than that of the control by 4.32% (Figure 5A). The total nitrogen content in both ammonium and nitrate nitrogen groups showed an upward trend with increasing nitrogen concentrations, where N3 and X3 displayed 28.4% and 48.9% higher total nitrogen content compared to the control group, respectively (Figure 5B). The intrinsic carbon and nitrogen content in plants is intrinsically linked to the C/N ratio balance, a key indicator of the relative demand for these elements across various plant organs. The *G. uralensis* root C/N ratio displayed markedly different responses to nitrogen forms, maintaining stability under ammonium treatments but exhibiting a non-monotonic pattern under nitrate treatments that first rose then fell with increasing concentration (Figure 5C).

**Figure 6:**
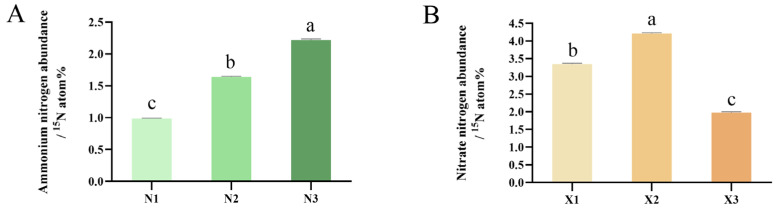
Content of ammonium nitrogen abundance (**A**) and ammonium nitrogen abundance (**B**) in the root of the *G. uralensis* with different nitrogen forms supply: 0.25, 0.5, 1.25 mmol/L (^15^NH_4_)_2_SO_4_ (treatments N1, N2, N3); 0.5, 1.0, 2.5 mmol/L K^15^NO_3_ (treatments X1, X2, X3). Different letters indicate a significant difference (*p* < 0.05).

Ammonium nitrogen isotope abundance in N1, N2, and N3 ranked as N3 > N2 > N1, with N2 and N3 being 1.66- and 2.25-fold higher than N1, respectively (Figure 6A). The nitrate nitrogen isotope abundance initially increased and then decreased from X1 to X3, peaking in the moderate concentration group (X2) at 1.26 times the level of X1. In contrast, the abundance in the high concentration group (X3) was significantly reduced to 0.59 times that of X1 (Figure 6B).

### 2.5. Total Proteins of G. uralensis Supplied Under Different Nitrogen Sources

**Figure 7:**
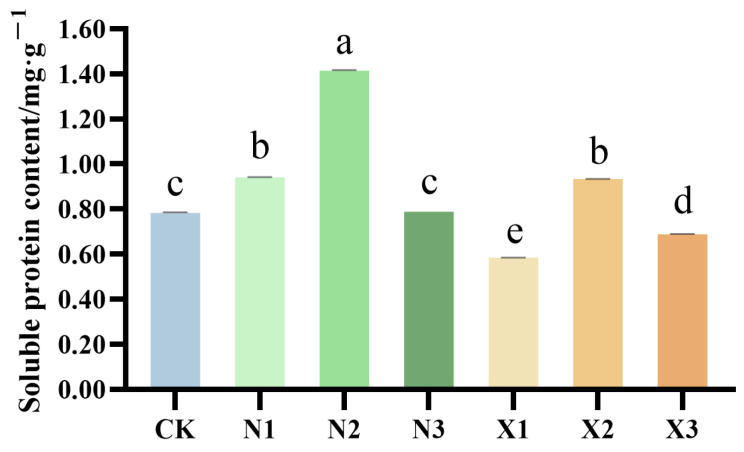
Content of soluble protein in the root of the *G. uralensis* with different nitrogen forms supply: modified Hoagland solution (control, CK); 0.25, 0.5, 1.25 mmol/L (^15^NH_4_)_2_SO_4_ (treatments N1, N2, N3); 0.5, 1.0, 2.5 mmol/L K^15^NO_3_ (treatments X1, X2, X3). Different letters indicate a significant difference (*p* < 0.05).

Soluble proteins are essential hydrophilic macromolecules in plants, acting as multifunctional osmotic regulators, enzymatic reserves, and transient nitrogen sinks that are central to nitrogen metabolism [21]. *G. uralensis* subjected to nitrogen treatments exhibited a biphasic response in soluble protein content, characterized by an initial increase followed by suppression. At equivalent nitrogen concentrations, ammonium-treated plants consistently accumulated higher soluble protein levels than those receiving nitrate. Specifically, the soluble protein content in groups N2 and X2 reached 180.40% and 119.09% of the control levels, respectively (Figure 7).

### 2.6. Amino Acid Contents of G. uralensis Under Different Nitrogen Treatments

**Figure 8:**
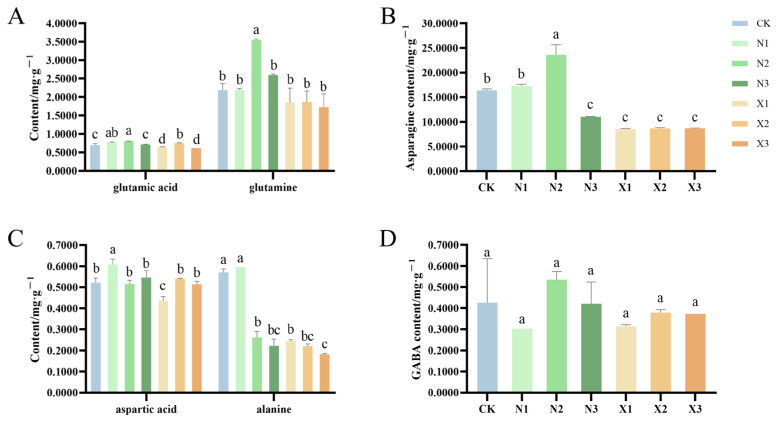
Contents of glutamic acid, glutamine (**A**), asparagine (**B**), aspartic acid, alanine (**C**), and GABA (**D**) in *G. uralensis* under different nitrogen forms: modified Hoagland solution (control, CK); 0.25, 0.5, 1.25 mmol/L (^15^NH_4_)_2_SO_4_ (treatments N1, N2, N3); 0.5, 1.0, 2.5 mmol/L K^15^NO_3_ (treatments X1, X2, X3). Different letters indicate statistically significant differences among treatments (*p* < 0.05).

Ammonium nitrogen supply resulted in elevated glutamic acid and glutamine contents in roots, whereas nitrate nitrogen treatments did not significantly affect glutamine levels (Figure 8A). Under ammonium nutrition, both glutamic acid and glutamine concentrations exhibited an initial increase followed by a decline as nitrogen application rates rose. Notably, the N2 treatment led to increases of 15.38% in glutamic acid and 61.79% in glutamine compared to the control. The asparagine content was higher under ammonium nitrogen than under nitrate nitrogen, with the highest accumulation observed in the N2 treatment, followed by N1 and N3 (Figure 8B). Specifically, the N2 treatment resulted in a significant increase of 43.88% in asparagine content compared to the control.

Except for the low ammonium nitrogen treatment, aspartic acid and alanine contents remained relatively consistent across nitrogen regimes (Figure 8C). The N1 treatment, however, resulted in a significant increase in aspartic acid content (16.48% higher than the control), along with a more modest rise in alanine (4.56% above control levels). The GABA content under both nitrogen forms showed a biphasic response to increasing supply rates, peaking at moderate levels before declining (Figure 8D).

### 2.7. Organic Acids Content of G. uralensis Supplied with Different Nitrogen Sources

**Figure 9:**
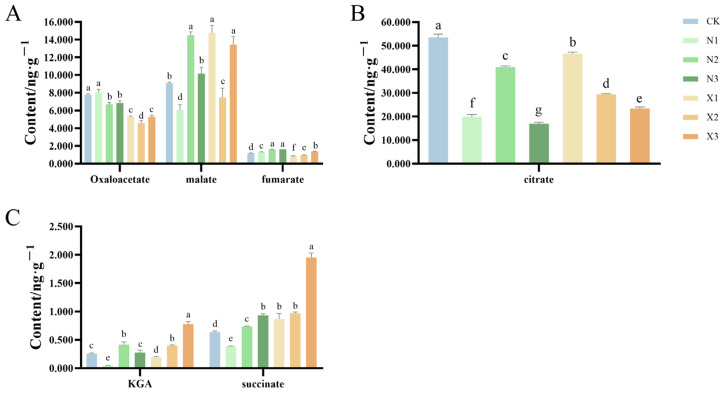
Contents of oxaloacetate, malate and fumarate (**A**), citrate (**B**), *α*-ketoglutarate (KGA) and succinate (**C**) in the root of the *G. uralensis* with different nitrogen forms supply: modified Hoagland solution (control, CK); 0.25, 0.5, 1.25 mmol/L (^15^NH_4_)_2_SO_4_ (treatments N1, N2, N3); 0.5, 1.0, 2.5 mmol/L K^15^NO_3_ (treatments X1, X2, X3). Different letters indicate a significant difference (*p* < 0.05).

Oxaloacetate, malate, and citrate exhibited distinct accumulation patterns under different treatments. Relative to the nitrate group, ammonium-treated plants showed elevated levels of oxaloacetate. Malate content was significantly elevated in groups N2, X1, and X3, with increases of 60.13%, 63.04%, and 48.11% compared to the control, respectively (Figure 9A). Among the ammonium nitrogen treatments, citrate levels were highest in N2, followed by N1 and N3, with N2 reaching 77% of the control value. Conversely, increasing nitrate nitrogen application was associated with a reduction in citrate accumulation in the nitrate-treated groups (Figure 9B).

Under ammonium nitrogen treatments, the accumulation of *α*-ketoglutarate (KGA) in *G. uralensis* exhibited a unimodal trend, reaching a maximum at the intermediate concentration (N2) with a significant increase of 62.97% compared to the control. In contrast, under nitrate nitrogen treatments, KGA content showed a positive linear dose–response relationship, with levels in X2 and X3 reaching 156.06% and 302.74% of the control values, respectively (Figure 9C). Both succinate and fumarate accumulated in a concentration-dependent manner across nitrogen regimens (Figure 9B,C). Notably, the X3 treatment showed the most pronounced accumulation of succinate, reaching 306.49% of the control level.

### 2.8. Activities of Enzymes Involved in N Metabolism of G. uralensis Supplied Under Different Nitrogen Sources

**Figure 10:**
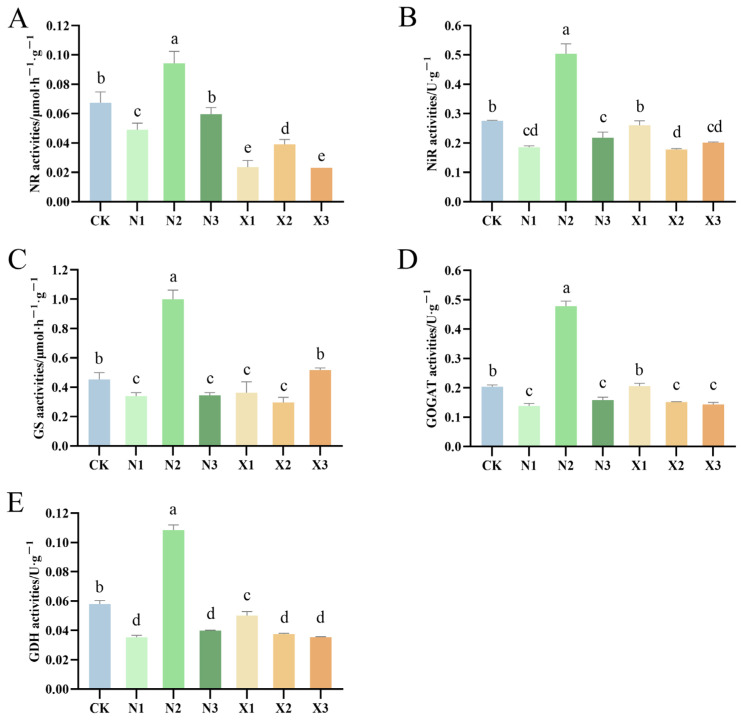
Nitrogen metabolism enzyme activities in *G. uralensis* roots in response to different nitrogen forms: modified Hoagland solution (control, CK); 0.25, 0.5, 1.25 mmol/L (^15^NH_4_)_2_SO_4_ (treatments N1, N2, N3); 0.5, 1.0, 2.5 mmol/L K^15^NO_3_ (treatments X1, X2, X3), showing (**A**) nitrate reductase (NR), (**B**) nitrite reductase (NiR), (**C**) glutamine synthetase (GS), (**D**) glutamate synthase (GOGAT), and (**E**) glutamate dehydrogenase (GDH). Different letters indicate a significant difference (*p* < 0.05).

Under ammonium nitrogen treatment, the activities of nitrate reductase (NR, EC 1.7.1.1), nitrite reductase (NiR, EC 1.7.2.1), glutamine synthetase (GS, EC 6.3.1.2), glutamate synthetase (GOGAT, EC 1.4.1.14), and glutamate dehydrogenase (GDH, EC 1.4.1.2) enzymes in *G. uralensis* exhibited a consistent trend, characterized by an initial increase followed by a decline with increasing ammonium nitrogen concentration (Figure 10). Compared to the control, a significant elevation in enzyme activities was observed under the N2 treatment, with marked increases of 40.10% (NR), 83.17% (NiR), 120.66% (GS), 134.86% (GOGAT), and 86.78% (GDH). In contrast, those enzymatic responses to nitrate nitrogen diverged. The activities of GOGAT and GDH were negatively correlated with the concentration of nitrate nitrogen in *G. uralensis*, whereas the activities of NiR demonstrated an initial decrease followed by an increase with increasing concentration.

**Figure 11:**
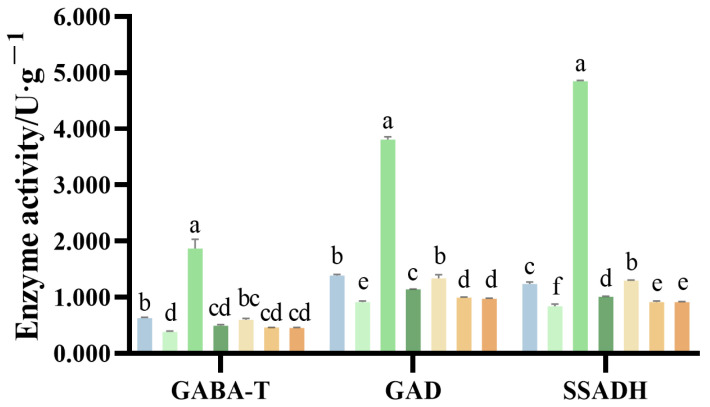
Activities of GABA metabolism-related enzymes in the *G. uralensis* with different nitrogen forms: modified Hoagland solution (control, CK); 0.25, 0.5, 1.25 mmol/L (^15^NH_4_)_2_SO_4_ (treatments N1, N2, N3); 0.5, 1.0, 2.5 mmol/L K^15^NO_3_ (treatments X1, X2, X3). Different letters above bars indicate statistically significant differences (*p* < 0.05).

In *G. uralensis* roots, the GABA shunt pathway initiates with the decarboxylation of glutamate to GABA, catalyzed by glutamate decarboxylase (GAD, EC 4.1.1.15). GABA is subsequently catabolized to succinate via the sequential actions of GABA transaminase (GABA-T, EC 2.6.1.19) and succinic semialdehyde dehydrogenase (SSADH, EC 1.2.1.24). As shown in Figure 11, ammonium nitrogen treatments elicited a concentration-dependent biphasic effect on the activities of GABA-T, GAD, and SSADH. Enzyme activities peaked at N2, reaching 296%, 275%, and 392% of the control levels, respectively. In contrast, nitrate nitrogen treatment resulted in a dose-dependent inhibition of these enzymes.

### 2.9. Transcript Levels of Key Genes Involved in N Metabolism of G. uralensis Supplied Under Different Nitrogen Sources

**Figure 12:**
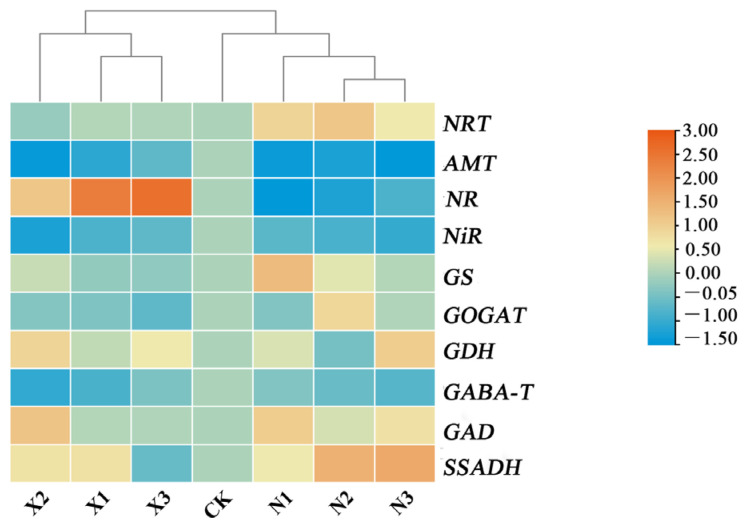
Hierarchical clustering of key nitrogen metabolism genes expression in *G. uralensis* under different nitrogen forms: modified Hoagland solution (control, CK); 0.25, 0.5, 1.25 mmol/L (^15^NH_4_)_2_SO_4_ (treatments N1, N2, N3); 0.5, 1.0, 2.5 mmol/L K^15^NO_3_ (treatments X1, X2, X3). For each gene, expression levels are normalized to the control group (set as 1). The color gradient (blue-yellow-red) in the figure indicates the relative gene expression levels, with warmer colors (closer to red) representing higher expression.

In the roots of *G. uralensis*, the genes *NRT*, *GS*, *GOGAT*, *GAD*, and *SSADH* exhibited greater responsiveness to ammonium nitrogen supply compared to nitrate nitrogen (Figure 12). The mRNA levels of *NRT, GS, GOGAT, GAD,* and *SSADH* were upregulated under ammonium nitrogen treatment, whereas their responses to nitrate nitrogen were variable. For instance, the expression of *NRT* and *GS* remained unaltered in response to nitrate nitrogen levels, while that of *GAD* was elevated under the X2 condition. In contrast, *NR* and *GDH* were upregulated under nitrate nitrogen supply. Additionally, the transcript levels of *SSADH* increased with rising ammonium nitrogen concentrations.

### 2.10. Correlation Analysis Between G. uralensis Respiratory and Primary Metabolism of Organic Acids and Amino Acids

**Figure 13:**
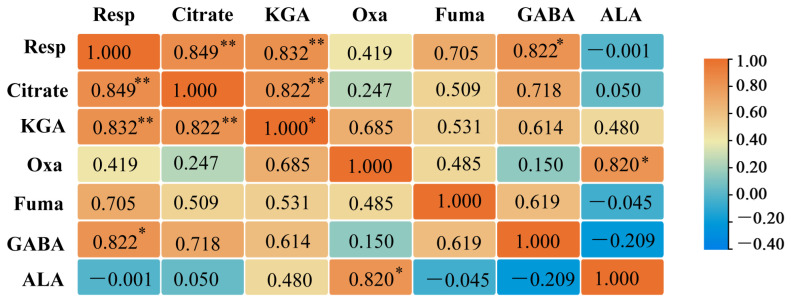
Heatmap of spearman correlations between respiration rate and metabolites (organic and amino acids) in *G. uralensis* under different nitrogen forms. Significance levels are indicated as follows: * *p* < 0.05, ** *p* < 0.01. Note: Resp: respiration rate, Oxa: oxaloacetate, Fuma: fumarate, ALA: alanine. The color gradient (blue-yellow-red) in the figure indicates a positive to negative correlation, with warmer colors (closer to red) representing positive correlation.

Spearman correlation analysis revealed a significant positive association between the root respiration rate of *G. uralensis* and citric acid, KGA, and GABA, with correlation coefficients (*r*) of 0.849, 0.832, and 0.822, respectively. Furthermore, a strong positive correlation was also identified between citrate and KGA (*r* = 0.822). Similarly, oxaloacetate and alanine showed a significant positive relationship, with a correlation coefficient of 0.820 (Figure 13).

### 2.11. Joint Analysis of the Effects of Different Forms of Nitrogen on Respiratory and Metabolism in G. uralensis

**Figure 14:**
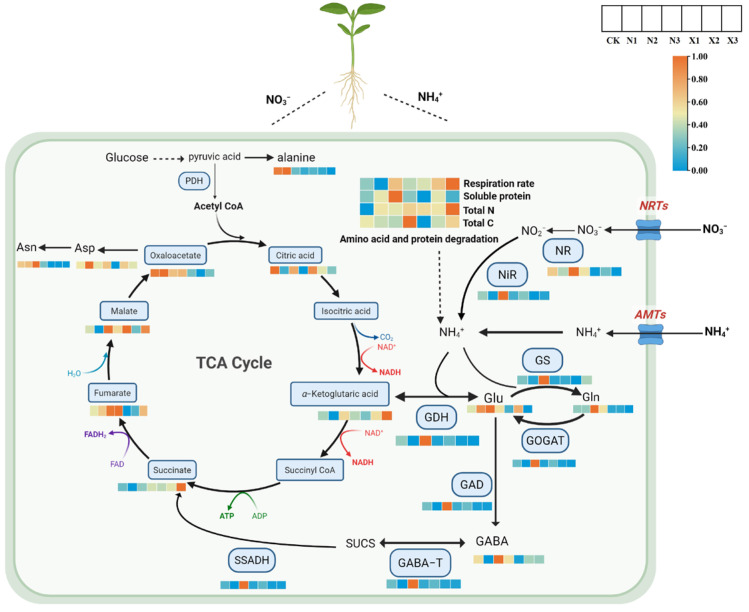
Profiling metabolites and key enzyme activities in nitrogen and amino acid pathways of *G. uralensis* in response to different nitrogen forms: modified Hoagland solution (control, CK); 0.25, 0.5, 1.25 mmol/L (^15^NH_4_)_2_SO_4_ (treatments N1, N2, N3); 0.5, 1.0, 2.5 mmol/L K^15^NO_3_ (treatments X1, X2, X3). The uptake of NH_4_^+^ and NO_3_^−^ into root cells is mediated by AMT and NRT transporters, respectively. While ammonium can be directly assimilated, nitrate is first reduced to ammonium by the sequential action of NR and NiR. Ammonium assimilation occurs primarily via the GS/GOGAT cycle, with GS incorporating NH_4_^+^ into Gln, while GDH provides a secondary pathway to Glu. The required α-ketoglutarate (KGA) is furnished by the TCA cycle—a central respiratory pathway that regenerates carbon skeletons through the sequential oxidation of citrate to KGA, succinate, fumarate, and malate. These nitrogen assimilation products are fundamental precursors for the synthesis of amino acids, proteins, and other growth-related metabolites. A color gradient (blue to red) represents the levels of key enzyme gene expression and metabolite abundance, with red indicating higher values normalized to the control group (set as 1). Note: glutamic acid (Glu), glutamine (Gln), aspartic acid (Asp) and asparagine (Asn).

Using TBtools, respiratory rate, respiratory metabolism-related organic acids, amino acids, total carbon, total nitrogen, soluble protein content, and nitrogen metabolism enzyme activities in *G. uralensis* were normalized and transformed. An integrated analysis was subsequently conducted to explore the nitrogen metabolism and amino acid biosynthesis pathways (Figure 14).

In plants, primary nitrogen metabolism encompassing nitrate reduction and subsequent assimilation is critical for growth and development. NR and NiR catalyze the stepwise reduction in nitrate to ammonium, which is then assimilated into glutamate and glutamine through the coordinated actions of GS, GOGAT and GDH. *G. uralensis* subjected to the N2 treatment exhibited a higher respiratory rate, along with increased activities of key nitrogen metabolic enzymes, including NR, NiR, GS, GOGAT and GDH, compared to the control. Consistent with these changes, the total nitrogen and soluble protein content were also significantly elevated in N2. Nitrogen assimilation relies on a source of KGA, which serves as the essential carbon acceptor in the GS/GOGAT pathway for the biosynthesis of glutamate-family amino acids. KGA is primarily supplied by the tricarboxylic acid (TCA) cycle—a central process in respiratory metabolism. The TCA cycle begins with the condensation of acetyl-CoA and oxaloacetate to form citrate. Through a series of four oxidative dehydrogenation reactions, coupled to the reduction in nicotinamide adenine dinucleotide (NAD^+^) and flavin adenine dinucleotide (FAD), and one substrate-level phosphorylation, the cycle sequentially produces KGA, succinate, fumarate, and malate. Finally, malate is oxidized by malate dehydrogenase, regenerating oxaloacetate to perpetuate the cycle. Notably, in the N2 treatment group, the levels of respiratory metabolism-related amino acids, glutamic acid (Glu), glutamine (Gln), and asparagine (Asn), and organic acids (citrate, KGA, fumarate, malate, and oxaloacetate) were significantly higher than those in the control group. The GABA shunt the bridges between N and C metabolism through TCA cycl. GABA is produced irreversibly by the cytosolic enzyme GAD and subsequently catabolized by GABA-T into succinic semialdehyde (SUCS), which is then converted by SSADH into succinate, thereby re-entering the TCA cycle [22]. The activities of enzymes associated with GABA metabolism, GAD, GABA-T, and SSADH were elevated in the N2 group relative to the control, along with increased levels of both GABA and SUS metabolites within the GABA shunt pathway. N2 suggests that ammonium nitrogen at optimal concentrations preferentially activates alternative NH_4_^+^ assimilation pathways, potentially enhancing TCA respiratory metabolism and nitrogen-use efficiency in *G. uralensis* roots.

Furthermore, the X3 treatment exhibited the highest respiratory rate, the greatest accumulation of respiratory metabolism-related organic acids (KGA and succinate), and the highest total nitrogen content. In contrast, the activities of key enzymes involved in both nitrogen metabolism and GABA metabolism were significantly reduced compared to the control. These results suggest that X3 enhances respiratory intensity and promotes the accumulation of organic acids such as KGA and succinate, while simultaneously suppressing nitrogen metabolic enzyme activity, ultimately leading to increased total nitrogen content in the metabolic products of *G. uralensis*.

## 3. Discussion

### 3.1. Nitrogen Absorption Characteristics and Their Relation to Nitrogen Metabolism Enzymes in G. uralensis

The differential uptake of ammonium (NH_4_^+^) and nitrate (NO_3_^−^), as a primary determinant of nitrogen assimilation strategy, activates distinct downstream metabolic networks in plants [23]. Our investigation, employing Non-invasive Micro-test Technology (NMT) to dynamically quantify ion fluxes, provided evidence for distinct absorption trends of *G. uralensis* roots in response to different nitrogen forms, suggesting potential unique uptake characteristics. Notably, the roots sustained a net NH_4_^+^ influx, the magnitude of which was positively correlated with the external NH_4_^+^ supply level. The metabolic interface for the absorbed NH_4_^+^ is predominantly the GS/GOGAT cycle. This cyclic pathway begins with the ATP-dependent amination of glutamate by glutamine synthetase (GS) to form glutamine. Glutamate synthase (GOGAT) subsequently catalyzes glutamine and KGA into two molecules of glutamate. One glutamate molecule is recycled within the cycle, while the other is diverted to the synthesis of proteins, nucleic acids, and other biomolecules. This coordinated mechanism, driven by the high NH_4_^+^ affinity of GS, ensures the swift and effective incorporation of absorbed ammonium into organic nitrogen compounds, thereby supporting the synthesis of proteins and other essential cellular constituents [24].

In ammonium-treated *G. uralensis* roots, GS mRNA levels were elevated in the N1 and N2 groups relative to the control. Notably, GS activity was comparatively higher in the N2 group, consistent with the enzyme’s high affinity for NH_4_^+^, which facilitates efficient function even at low substrate concentrations. In contrast, GOGAT activity in ammonium-treated roots displayed a non-linear response, increasing initially, then declining with rising ammonium supply. Specifically, the N2 group exhibited a 134.86% increase in GOGAT activity over the control, likely due to the NH_4_^+^-induced upregulation of GOGAT gene expression at moderate NH_4_^+^ levels [25]. This coordinated induction of GS and GOGAT suggests that the N2 treatment optimally promotes NH_4_^+^ assimilation. Meanwhile, glutamate dehydrogenase (GDH), which operates significantly under medium NH_4_^+^ concentrations due to its lower substrate affinity, also showed elevated activity, likely supporting NH_4_^+^ assimilation in response to sustained root-level ammonium availability.

The net influx of NO_3_^−^ in *G. uralensis* root tips transitioned from absorption to efflux trend as nitrate concentration increased. Nitrate assimilation is initiated by nitrate reductase (NR), a rate-limiting inducible enzyme that consumes reductants (NADH or NADDPH) and protons [26]. Yet our data indicate that downstream processes governed the demand for NO_3_^−^ uptake. Among nitrate treatments, we observed the highest NR activity in X2, whereas nitrite reductase (NiR) activity was greatest in X1. The most substantial net NO_3_^−^ influx occurred in the X1 treatment, likely due to the concerted high activity of NiR and the complementary activities of GOGAT and GDH. The observed profile may indicate that X1 roots appear to process absorbed NO_3_^−^ more effectively through its reduction and subsequent assimilation, potentially supporting a greater metabolic demand and sustained influx. The decline in NR activity at the highest nitrate level further correlates with the reduced influx, indicating a feedback regulation of nitrate uptake.

### 3.2. Utilization Characteristics of Ammonium and Nitrate Nitrogen in G. uralensis Roots

^15^N isotope tracing data suggested an apparent divergence in nitrogen utilization patterns within *G. uralensis* roots across the different nitrogen forms supplied. For ammonium, root ^15^N abundance showed a positive correlation with external concentration, evidenced by a 124.92% greater value in N3 than in N1 roots, suggesting a high capacity for NH_4_^+^ uptake and assimilation. For nitrate, however, root ^15^N abundance peaked at intermediate concentrations (X1/X2) and decreased at the highest level (X3). This finding was consistent with the dynamic NO_3_^−^ absorption profiles obtained via NMT. These results collectively suggest a plausible physiological framework wherein moderate nitrate levels are conducive to efficient utilization, whereas excessive levels could downregulate the assimilation pathway, potentially saturating its capacity or inducing feedback inhibition that might impair subsequent uptake or assimilation [27].

### 3.3. Relationship Between Nitrogen Absorption/Utilization and Respiratory Metabolism in G. uralensis

Root respiration, consuming up to 75% of belowground carbon, provides the essential energy for growth and nutrient acquisition, thereby significantly influencing plant primary productivity [28]. In this study, root respiration in *G. uralensis* exhibited a distinct biphasic response to ammonium (NH_4_^+^), increasing initially then decreasing, whereas it showed a steady, concentration-dependent increase in response to nitrate (NO_3_^−^). Plant root respiration is influenced by multiple soil factors, including temperature and nutrient availability, among which nitrogen is a key regulator [29]. Our earlier study showed that nitrogen and respiratory metabolism jointly regulate the growth and accumulation of active components in *G. uralensis* [4,5]. Given the pivotal role of the TCA cycle in this respiratory process, changes in respiratory intensity and pathway inevitably impact the types and levels of resulting metabolic intermediates.

Our findings demonstrate that differential nitrogen form utilization in *G. uralensis* regulates the key enzymes of nitrogen assimilation (NR/NiR and GS/GOGAT) at both transcriptional and activity levels, with downstream effects on the accumulation of glutamate and glutamine. Furthermore, glutamate is channeled into the GABA shunt by GAD, and GABA is further metabolized by GABA-T and SSADH to generate succinate for the TCA cycle. In this study, under ammonium treatment, the contents of glutamate and GABA, along with the activities of GAD, GABA-T, SSADH, and the respiratory rate, all exhibited similar non-linear trends. The consistent patterns observed for glutamate, glutamine, GABA, and succinate demonstrate a highly coordinated physiological response that integrates GABA metabolism with central nitrogen and respiratory metabolism. Despite enhanced ammonium absorption at the N3 level, *G. uralensis* roots showed suppressed metabolism. A decline in GOGAT activity reduced glutamate availability, which in turn downregulated the GABA shunt, as evidenced by decreased activities of GAD, GABA-T, and SSADH. The consequent drop in succinate supply attenuated the respiratory metabolism, ultimately leading to lower overall respiratory intensity in the N3 treatment compared to N2.

Furthermore, as the ammonium nitrogen concentration increased, the levels of key TCA cycle intermediates (citric acid, KGA, and malic acid) in ammonium-treated plants exhibited a biphasic response, rising initially before declining. Concomitantly, the abundance of nitrogen assimilation products (glutamine, asparagine, and soluble protein) mirrored the changes in respiratory intensity. These coordinated patterns indicated that low-to-moderate ammonium levels enhance both respiratory activity and nitrogen metabolite accumulation in *G. uralensis*. This optimal activation likely stems from the plant’s assimilation of absorbed NH_4_^+^, a process that demands carbon skeletons and energy supplied by respiratory metabolism [30]. Such a metabolic coupling would thereby promote a synergistic enhancement between the TCA cycle and nitrogen assimilation pathways.

In nitrate-treated *G. uralensis*, metabolites of the glutamate-GABA pathway (KGA and succinate) increased with nitrate supply, paralleling the rise in respiratory intensity. In contrast, nitrogen assimilation products (e.g., soluble protein) and the C/N ratio followed a biphasic pattern, peaking at moderate levels before declining. This indicated that carbon metabolism initially predominates, fueling respiration and the accumulation of glutamate and GABA under low-to-moderate nitrate. At the highest nitrate level (X3), however, a metabolic shift occurred, characterized by reduced NO_3_^−^ uptake and weakened carbon metabolism while nitrogen assimilation intensified. Under these conditions, the accelerated catabolism of glutamate and GABA is likely compensated by replenishing KGA and succinate to the TCA cycle, thereby playing a critical role in sustaining respiratory energy production under high nitrogen load.

## 4. Materials and Methods

### 4.1. Plant Growth Conditions and Treatments

*G. uralensis* seeds were obtained from China National Traditional Chinese Medicine Co., Ltd. (Beijing, China). These seeds were sown in hydroponics rectangular pots (upper length 29 cm, width 23 cm, lower length 23 cm, width 18 cm, and depth 6 cm) in an artificial climate chamber. Seeds were grown in hydroponics pots filled with 500 mL pure water at a photoperiod of 12 h, temperature 25 ± 1 °C, humidity 60 ± 5%, dark period 12 h, temperature 20 ± 1 °C, humidity 40 ± 5%. Upon emergence of the first true leaf (day 9), the plants were transferred to and cultivated in 500 mL modified Hoagland nutrient solution (HNS, 0.44 g/L CaCl_2_, 0.493 g/L MgSO_4_·7H_2_O, 0.136 g/L KH_2_PO_4_, 2.86 mg/L H_3_BO_3_, 1.81 mg/L MnCl_2_·4H_2_O, 0.22 mg/L ZnSO_4_·7H_2_O, 0.08 mg/L CuSO_4_·5H_2_O, 0.03 mg/L Na_2_MoO_4_·2H_2_O, 5.56 mg/L FeSO_4_·7H_2_O, 7.46 mg/L EDTA, PH = 7.4) at photoperiod 14 h with temperature 25 ± 1 °C and humidity 60 ± 5%, dark period 10 h with temperature 20 ± 1 °C and humidity 40 ± 5%. 0.25 mmol/L ammonium (^15^NH_4_)_2_SO_4_, 0.5 mmol/L ammonium (^15^NH_4_)_2_SO_4_, 1.25 mmol/L ammonium (^15^NH_4_)_2_SO_4_, 0.5 mmol/L nitrate (K^15^NO_3_), 1 mmol/L nitrate (K^15^NO_3_), and 2.5 mmol/L nitrate (K^15^NO_3_) were separately dissolved in modified HNS. After the emergence of two true leaves (day 14), the uniformly growing *G. uralensis* seedlings were randomly assigned to different nitrogen nutrient solutions, and presented as 0.25 mmol/L (^15^NH_4_)_2_SO_4_ (low ammonium, N1), 0.5 mmol/L (^15^NH_4_)_2_SO_4_ (medium ammonium, N2), 1.25 mmol/L (^15^NH_4_)_2_SO_4_ (high ammonium, N3), 0.5 mmol/L K^15^NO_3_ (low nitrate, X1), 1 mmol/L K^15^NO_3_ (medium nitrate, X2), 2.5 mmol/L K^15^NO_3_ (high nitrate, X3) and modified HNS treatment group (Control, CK). Following a 6-day ^15^N tracer treatment (day 20), *G. uralensis* seedlings from each group were biologically sampled in triplicate. A portion of the samples was subjected to Non-invasive Micro-test Technology (NMT) measurements; the remainder was immediately snap-frozen in liquid nitrogen and stored at −80 °C for analysis of physiological and molecular characteristics. In each group, representative plants were used to measure growth parameters.

### 4.2. Measurement of Plant Biomass

Following harvest, plants from each group were separated into roots and shoots. The length of each organ was measured, and its dry weights were determined after oven-drying.

### 4.3. Measurement of the Rate of Respiration

The root was washed with pure water and wrapped with wet gauze, and then washed twice with a neutral detergent prior to being immersed in a 3% H_2_O_2_ solution to minimize interference from root-associated microorganisms. The rate of root respiration was measured according to the previously method [4]. Briefly, the root was subsequently transferred to a reaction vessel containing 9 mL of 0.2 M Tris-HCl buffer (pH 7.4). Root respiration rate was measured at 25 °C using a Yaxin-1151 Biological Oxygen Analyzer (Yaxinliyi Science & Technology Co., Ltd., Beijing, China), whereby each sample was measured for 5 min with six replicates per group.

### 4.4. Determination of Net Fluxes of NH_4_^+^ and NO_3_^−^

The net fluxes of NH_4_^+^ and NO_3_^−^ in the root meristematic zone were measured using the Non-invasive Micro-test Technology (NMT, YoungerUSA LLC, Amherst, MA, USA) as described in a previous study [31,32]. Briefly, Pre-pulled and silanized microsensors were first filled with a backfilling solution (100 mM NH_4_Cl for the NH_4_^+^ electrode, 10 mM KNO_3_ for the NO_3_^−^ electrode). The micropipettes were front-filled with 40–80 μm columns of selective liquid ion-exchange cocktails. An Ag/AgCl wire microsensor holder XY-CGQ01 (YoungerUSA, Amherst, MA, USA) was inserted in the back of the microsensor. Before the flux measurements, the ion-selective electrodes were calibrated by a series of standard solutions (Xuyue Company, Beijing, China). The fine seedling roots were equilibrated for 20 min in measuring solution (0.1 mM CaCl_2_, 0.3 mM MES, pH 6) with 0.5, 1, or 2.5 mM NH_4_NO_3_ according to the nitrogen treatment of the selected roots. With three replicates per treatment group, real-time influxes of NH_4_^+^ and NO_3_^−^ were monitored at a point approximately 5 μm above the root meristem surface, with a 10-min recording per sample.

### 4.5. Determination of Content of Total N, Total C, δ^15^N and Total Proteins

δ^15^N, total N and total C concentrations in the roots were obtained as proposed by Sen Meng et al. [8]. An appropriate amount of dried sample was sealed in a tin cup. Then the isotopic ratio, total N and total C concentration were analysed using an elemental analyser (EA IsoLink CN/OH) coupled with a Delta V Advantage gas isotope ratio mass spectrometer (Thermo Corporation, Waltham, MA, USA).

The contents of total soluble protein in the sample root were determined using the Coomassie Brilliant Blue method [33].

### 4.6. Determinations of Amino Acids, Organic Acids

Dry root samples (0.2 g) were extracted with 10 mL boiling water and water bath at 80 °C for 5 min, after centrifugation at 6000 r/min for 10 min, 1 mL supernatant was mixed with 0.5 mL 0.1 mol/L Na_2_B_4_O_7_ and 0.5 mL 1% DNFB solution in tube, reacting in a 60 °C water bath in the dark for 1 h, cool to room temperature, dilute with 0.02 mol/L K_2_HPO_4_ and 0.02 mol/L KH_2_PO_4_ to 5 mL, then the mixture was filtered (0.22 μm pore size) to vials for amino acids analysis. The content of the main amino acids (aspartic acid, glutamic acid, glutamine, GABA, alanine, asparagine) of the samples was analysed using a Shimadzu LC-20AT HPLC system. A DIKMA Diamonsil AAA C_18_ column (4.6 mm × 250 mm, 5 μm) was utilized for chromatographic separation. The mobile phase comprised water (A, 0.02 mol/L K_2_HPO_4_ + 0.02 mol/L KH_2_PO_4_) and 10% methanol-90% acetonitrile (B) followed at 0.8 mL/min in a gradient elution (0~9 min, 14%~20% B; 9~10 min, 20%~21% B; 10~39 min, 21%~40% B; 39~44 min, 40%~70% B; 44~44.01 min, 70%~14% B; 44.01~60 min, 14%~14% B). The column temperature was maintained at 45 °C. An injection volume of 10 μL was used for each sample. The detection wavelength was set at 360 nm.

For organic acids analysis, fresh root samples were mixed with pure water and ground to obtain a 100 mg/mL homogenate solution. Then transfer the sample solution to a centrifuge tube, shaking for 1 min before being centrifuged at 12,000 rpm for 5 min at 4 °C. The supernatant solution was then filtered with a 0.22 μm pore size membrane and stored in chromatographic sample bottles. Then, the sample extracts were analyzed in negative ionization mode on a UPLC system (Acquity 1 class PLUS Waters) equipped with a Waters Xevo TQ-Smicro triple quadrupole mass and a Acquity UPLC BEH C_18_ (1.7 µm, 2.1 mm × 100 mm) column under the following conditions: mobile phase consisting of pure water with 0.5% formic acid (A) and acetonitrile (B), gradient program: 0~0.5 min, 5%~5% B; 0.5~0.6 min, 5%~30% B; 0.6~3 min, 30%~40% B; 3~3.1 min, 40%~100% B; 3.1~7 min, 100%~100% B; 7~7.1 min, 100%~5% B; 7.1~10 min, 5%~5% B, flow rate: 0.3 mL/min, the column oven temperature: 50 °C, injection volume: 3 μL. The triple quadrupole (QQQ) scans were acquired on a multi-reaction monitoring (MRM) mode and controlled by MassLynx 4.1 software.

### 4.7. Determinations of Activities of Enzymes Involved in N Metabolism

Glutamine synthetase activity (GS) and Nitrate reductase activity (NR) in roots were measured according to the instructions provided by the manufacturer of the assay kit (JCbio, Nanjing, China). Nitrite reductase activity (NiR), Succinate semialdehyde dehydrogenase activity (SSADH), Gamma aminobutyric transaminase activity (GABA-T), Glutamate decarboxylase activity (GAD), Glutamate dehydrogenase activity (GDH) and Glutamate synthase activity (GOGAT) in roots were measured by the ELISA assay kit’s instructions (YJbio, Shanghai, China).

### 4.8. Analysis of the Transcript Levels of Key Genes Involved in N Metabolism

Total RNA was extracted from root samples using an RNAprep Pure Plant Kit (DP432, Tiangen, China) according to the manufacturer’s instructions. RNA quality was evaluated by measuring the OD260/OD280 ratio and by electrophoretic analysis. The purified RNA was reverse-transcribed into first-strand cDNA using a PrimeScript^TM^ cDNA Reverse Transcription Reagent Kit with gDNA Eraser (Takara, RR047A, Kusatsu, Shiga, Japan). Quantitative real-time PCR (qRT-PCR) was subsequently performed on a CFX96 Real-Time PCR System (Bio-Rad, Hercules, CA, USA) using Taq Pro Universal SYBR qPCR Master Mix (Vazyme, Nanjing, China). The primers of target N metabolism genes (*NR/NiR*, *GS/GOGAT*, *GDH*, *GAD*, *SSADH*, *GABA-T*, *NRT*, *AMT*) used for the qRT-PCR were based on our previous research [5]. The 2^−ΔΔCt^ method was used to calculate the relative transcript levels.

### 4.9. Statistical Analysis

The net flux results were calculated with the MageFlux program of the NMT system (http://www.xuyue.net/mageflux, accessed on 22 December 2025). Statistical analyses were performed using SPSS 20.0 (SPSS Inc., Chicago, IL, USA). Differences among groups were evaluated by one-way ANOVA, followed by the Student-Newman-Keuls (SNK) test for multiple comparisons. A * *p*-value of <0.05 was considered statistically significant. The correlation between respiration rate and respiratory metabolism was assessed using Spearman’s rank correlation analysis in SPSS.

Cluster and Correlation Heatmap Analysis: gene expression data were log-transformed and normalized to a [0, 1] scale by row. Hierarchical cluster analysis was then performed using the TBtools-II software (v2.332) with the following parameters: a distance matrix based on Euclidean distance and clustering via the complete linkage method; results are presented as a cladogram. Furthermore, a heatmap visualizing the Spearman correlation coefficients was generated using TBtools-II with no additional scaling and a round-rectangle tile shape.

## 5. Conclusions

In conclusion, this study elucidates the integrated response of *G. uralensis* to different nitrogen forms, revealing an interconnected modulation of its physiology, respiratory metabolism, and primary nitrogen assimilation. *G. uralensis* employs distinct nitrogen uptake and utilization strategies, dictated by the form and concentration of nitrogen available. Integrative data from both NMT and ^15^N tracing revealed that *G. uralensis* displayed distinct utilization efficiencies for the two nitrogen forms and that it efficiently absorbed and utilized ammonium at optimal levels and nitrate at low-to-moderate concentrations. In contrast, a high nitrate supply suppressed both nitrate uptake and utilization. And consequently modulated nitrogen metabolism, as manifested by altered activities of nitrogen-assimilating enzymes, fluctuations in glutamate levels, and differential regulation of key enzymes in the endogenous GABA shunt. While ammonium nitrogen more potently enhanced the primary ammonia assimilation and GABA pathways, leading to higher accumulation of glutamate and endogenous GABA, nitrate nitrogen exerted a stronger stimulatory effect on the respiratory metabolites KGA and succinate. Furthermore, the nitrogen-induced enhancement of respiratory metabolism was accompanied by an accelerated catabolism of endogenous GABA, which thereby replenished the consumed KGA in the respiratory pathway.

## Data Availability

The original contributions presented in this study are included in the article/Supplementary Materials. Further inquiries can be directed to the corresponding author.

## Supplementary Materials

The following supporting information can be downloaded at https://www.mdpi.com/article/10.3390/ijms27010317/s1.

## Abbreviations

The following abbreviations are used in this manuscript:

NRT	Nitrate transporters
NR	Nitrate reductase
NiR	Nitrite reductase
GS	Glutamine synthetase
GOGAT	Glutamate synthetase
GDH	Glutamate dehydrogenase
AMTs	Ammonium transporters
Gln	Glutamine
Glu	Glutamic acid
KGA	*α*-ketoglutarate
TCA cycle	Tricarboxylic acid cycle
Oxa	Oxaloacetate
GABA	gamma-Aminobutyric acid
GAD	Glutamate decarboxylase
GABA-T	GABA transaminase
SUCS	Succinic semi-aldehyde
SSADH	Succinic semi-aldehyde dehydrogenase
PDH	Phosphofructokinase
Asn	Asparagine
Asp	Aspartic acid
