# Supplementary material for: Integration of Non-Invasive Micro-Test Technology and 15N Tracing Reveals the Impact of Nitrogen Forms at Different Concentrations on Respiratory and Primary Metabolism in Glycyrrhiza uralensis
Source: Int J Mol Sci. 2025 Dec 27;27(1):317. doi: 10.3390/ijms27010317 (PMC12786065; doi:10.3390/ijms27010317)
Supplement: Supplementary file 1 [file ijms-27-00317-s001.zip › ijms-4027986-supplementary.pdf]

Supplementary Materials: Integration of Non-Invasive Micro-Test Technology and ^{15}N Tracing Reveals the Impact of Nitrogen Forms at Different Concentrations on Respiratory and Primary Metabolism in *Glycyrrhiza uralensis*

Ying Chen [1,2], Yisu Cao [1], Yuan Jiang [1], Yanjun Wang [1], Zhengru Zhang [1], Yuanfan Zhang [1] and Zhirong Sun [1,*]

1 School of Chinese Materia Medica, Beijing University of Chinese Medicine, Beijing 102488, China

2 Faculty of Pharmacy, Fujian University of Traditional Chinese Medicine, Fuzhou 350122, China

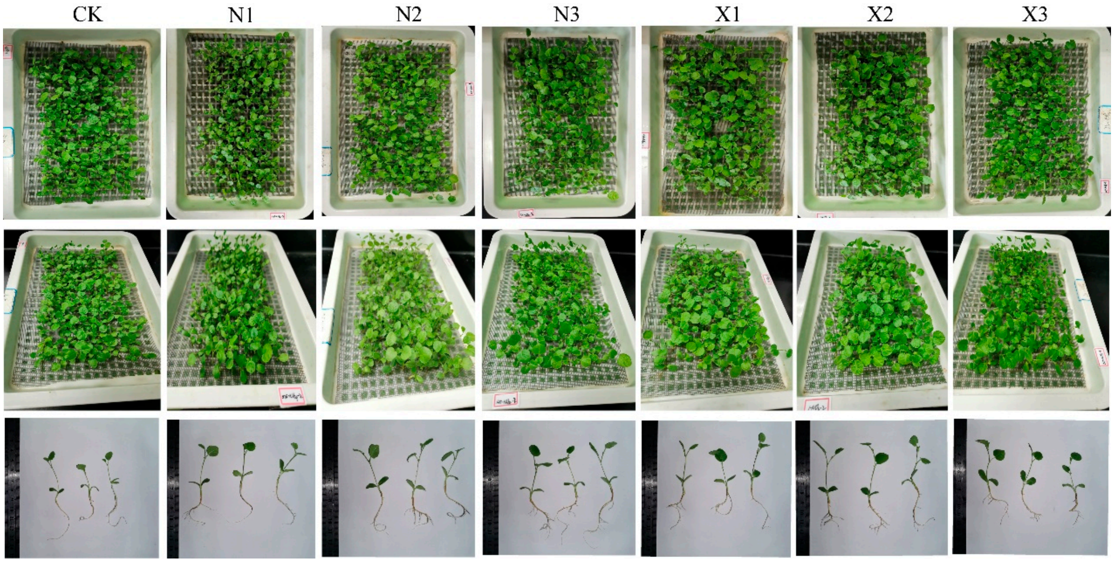

Figure S1 Phenotype characteristics of *G. uralensis* exposed to different nitrogen sources and concentrations. Seedlings were treated as follows: modified Hoagland nutrient solution (Control, CK); ammonium: 0.25,0.5,1.25 mmol/L $(^{15}NH_4)_2SO_4$ (labeled N1, N2, and N3, respectively); nitrate: 0.5,1,2.5 mmol/L $K^{15}NO_3$ (labeled X1, X2, and X3, respectively).
